# Case report: The CT features of pediatric retroperitoneal extrarenal Wilms tumor: a report of two cases and literature review

**DOI:** 10.3389/fped.2023.1161603

**Published:** 2023-05-23

**Authors:** Ting Li, Haoru Wang, Xin Chen, Ling He

**Affiliations:** Department of Radiology, Children’s Hospital of Chongqing Medical University, National Clinical Research Center for Child Health and Disorders, Ministry of Education Key Laboratory of Child Development and Disorders, Chongqing Key Laboratory of Pediatrics, Chongqing, China

**Keywords:** children, extrarenal Wilms tumor, computerized tomography, case report, literature review

## Abstract

Retroperitoneal extrarenal Wilms tumor is a rare condition in children that can be easily misdiagnosed as other retroperitoneal malignancies unrelated to the renal origin. Computerized tomography scan plays a crucial role in diagnosing and distinguishing retroperitoneal malignancies. In this report, we present two cases of retroperitoneal extrarenal Wilms tumor in children who were admitted due to abdominal mass. Laboratory examination did not reveal any significant abnormality. However, a computerized tomography scan revealed a solid or cystic-solid mass in the retroperitoneum accompanied by a bone spur extending from the anterior edge of the vertebral body to the back of the mass, while the origin of the tumor remained unclear. By analyzing these two cases and reviewing previous studies on retroperitoneal extrarenal Wilms tumor in children, we summarized the clinical and imaging characteristics of this rare condition. We also found that the presence of a spinal deformity adjacent to the mass might indicate the possibility of a retroperitoneal extrarenal Wilms tumor.

## Introduction

Extrarenal Wilms tumor (EWT) is a rare type of Wilms tumor (WT) in children and accounts for approximately 3% of WT (1). It is most common in the retroperitoneum, followed by the inguinal area, scrotum, and other locations. EWT and WT have similar histological characteristics and usually use the same staging and treatment principles internationally, but EWT may originate from primitive mesonephros or pro-kidney residues, which are more primitive than intrarenal WT. The clinical symptoms and signs of EWT often lack specificity, and most preoperative imaging studies are limited to case reports. To address this issue, we seek to combine the imaging features of pediatric retroperitoneal EWT observed in our hospital with previous literature to summarize the clinical and imaging findings, as well as unique signs that can aid in diagnosis.

## Case description

**Figure 1:**
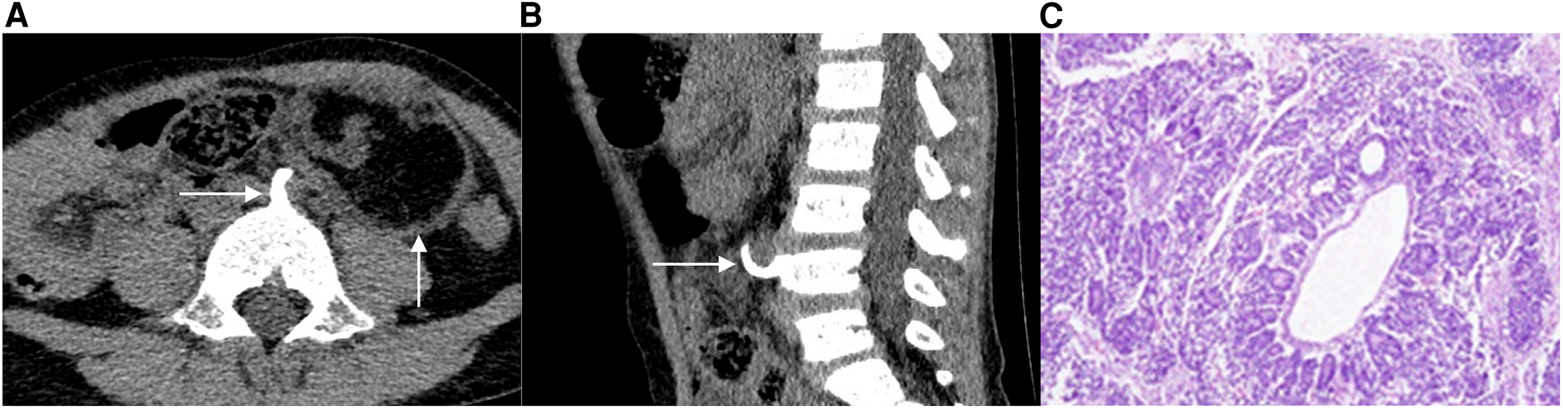
A 4-year-old female patient with EWT. The CT scan in a transverse position reveals the presence of an oval mixed-density mass in the left middle and lower abdomen, with a bone spur extending from the front of the lumbar fourth vertebra to the back of the mass (1**A**). In the sagittal position, the CT scan shows a slight collapse of the bone in the anterior part of the lumbar fourth vertebrae and the presence of a bone spur on the anterior edge (1**B**). Additionally, image C displays the 100× hematoxylin-eosin (HE) staining of the tumor, revealing the presence of multilayered differentiated tumors (1**C**).

**Case 1** A 4-year-old female patient was admitted to the hospital after the discovery of an abdominal mass. There was no discomfort, and laboratory examination revealed no abnormalities; a computerized tomography (CT) scan revealed an oval mixed-density mass in the left middle and lower abdomen with a clear boundary. The dimensions of the mass measured on the image were approximately 51 mm in length, 39 mm in width, and 52 mm in height. The mass was primarily composed of fat density (Figure 1A), with a small amount of punctate calcification at the edge, and showed slight enhancement after the enhancement process. Moreover, the fourth lumbar vertebral body's anterior portion had collapsed, and a bone spur had formed at the anterior edge, growing forwards and upwards through the beginning of the left common iliac artery before finally reaching the back of the mass (Figure 1B). The left kidney was positioned low, with its upper pole located around the level of the second lumbar vertebra body. Furthermore, an accessory renal artery was observed entering the left renal artery from the beginning of the left iliac artery. During the operation, a cystic solid mass containing hair and sebaceous substances was discovered at the end of the left descending colon. The pathological examination revealed the presence of multilayered differentiated tumors, primarily composed of nephroblastoma components with germ-like structures and a small number of tubule-like structures, adipose tissue, glandular epithelial differentiation, and localized myxoid degeneration (Figure 1C). The pathological diagnosis confirmed the presence of WT with heterogeneous component differentiation.

**Figure 2:**
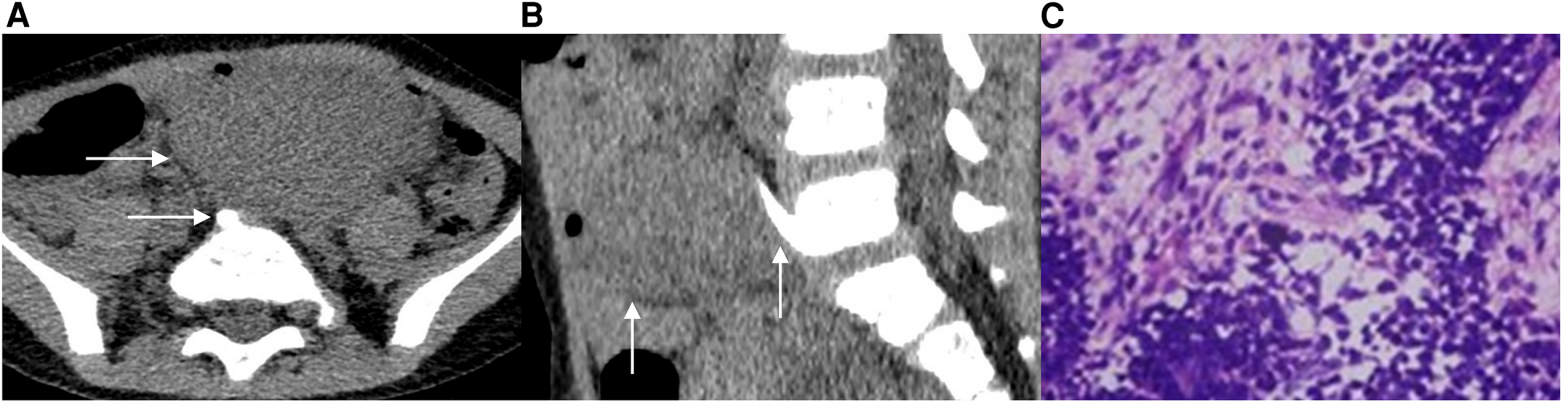
A 3-year-old male patient with EWT. In the CT transverse position, a soft tissue mass is visible behind the left peritoneum. The mass extends across the midline and features a bone spur located at the front of the fifth lumbar vertebrae body, with its tip reaching the posterior edge of the mass (2**A**). In the sagittal position, the CT scan shows the presence of a bone spur that originated from the anterior edge of the L5 vertebral body and grew forward and upward to the posterior edge of the mass (2**B**). Additionally, image C displays the 100× hematoxylin-eosin (HE) staining of the tumor, revealing the presence of epithelial tumor cells and mesenchymal tumor cells (2**C**).

**Case 2** A 3-year-old male patient was admitted to the hospital after the discovery of an abdominal mass. No discomfort was reported, and laboratory examination showed no apparent abnormality. A CT scan revealed the presence of a soft tissue mass with a clear boundary in the left retroperitoneum. The mass measured approximately 38 mm in length, 64 mm in width, and 44 mm in height in the image (Figure 2A). After the contrast-enhanced scan, the mass displayed inhomogeneous enhancement with dotted and striped vascular shadows. Additionally, a bone spur was observed originating from the anterior edge of the fifth lumbar vertebral body, growing forward and upward to the posterior edge of the mass (Figure 2B). During the operation, a solid mass was discovered in front of the bifurcation of retroperitoneal vessels, with abundant blood supply and an incomplete capsule. The pathological examination revealed the presence of germ-type tumor cells distributed in flakes, accounting for approximately 50%–60% of the tumor tissue area, with visible pathological nuclear fission images. Furthermore, epithelial tumor cells were arranged in small tubules, accounting for about 30%–40% of the tumor tissue area, while a few mesenchymal tumor cells were observed, accounting for approximately 5%–10% of the tumor tissue area (Figure 2C). The final pathological diagnosis confirmed the presence of retroperitoneal WT with focal degeneration.

## Discussion

WT is a prevalent malignant tumor in children, comprising around 7% of all malignant tumors in this age group (2). EWT, on the other hand, is a rare form of WT, representing approximately 3% of all cases (1). The majority of previous studies on EWT have been limited to case reports (3). EWT is most often located in the retroperitoneum, followed by the inguinal, scrotum, uterine cavity, lumbosacral subcutaneous, bladder, repeated sigmoid colon, and other locations (4, 5). EWT located in the retroperitoneal region commonly manifests as clinical symptoms including abdominal mass, pain, and compression of adjacent organs (6). While a preoperative CT scan can aid in the detection of retroperitoneal tumors, accurately diagnosing EWT using this imaging method is challenging due to the absence of distinctive CT signs. As such, postoperative pathological examination remains the gold standard for EWT diagnosis (7).

We conducted a search for reports of retroperitoneal EWT that included detailed CT data or CT images between 1990 and 2022. Our search yielded a total of eight cases of retroperitoneal EWT in children, reported in previous works of literature. The proportion of male and female patients was similar, with six male patients and four female patients among the 10 cases. The age of the patients ranged from 8 days to 17 years, with a median age of 3 years. Notably, 80% of the cases were observed in children less than 5 years old. All 10 cases were unilateral, with most of the tumors located around the midline. There was no significant difference in the incidence of EWT between the left and right sides, which is consistent with previous literature.

EWT primarily comprises germ tissue, epithelial cells, and interstitial components, with varying proportions of each component (8). The interstitial component is mainly composed of myofibroblast-like spindle cells, which have the ability to differentiate into various heterogeneous components. Most commonly, these cells differentiate into striated muscle but can also differentiate into other tissues such as smooth muscle, fat, bone, cartilage, and nerve tissue (9).

The most common CT findings of EWT are solid or cystic-solid masses, with round or oval shapes and some lobulated shapes. These masses are typically larger in size and may extend across the midline, compressing surrounding tissues and large blood vessels. Density may be uneven in the presence of fat or calcification within the mass (Table 1). In Case 1, the CT scan revealed an oval mixed cystic-solid mass located in the left middle and lower abdomen, with a clear boundary. The mass exhibited fat density and calcification with imaging features that can be easily mistaken for immature teratoma. Therefore, pathological examination remains the gold standard for EWT diagnosis (10).

**Table 1:** Patients’ general information and imaging characteristics.

Gender	Age	Site	Imaging features					Author
			Shape	Across the midline	Lump property	Heterogeneous	Long diameter (mm)	
Male	8 days	Right	Lobulated	Yes	Solid	Yes	100	Yunus et.al. (11)
Female	5 years	Right	Lobulated	Yes	Cystic solid	No		Arda et al. (7)
Male	17 years	Left	Circle or ellipse	No	Solid	No	120	Gordetsky et al. (12)
Female	2 years	Left	Circle or ellipse	Yes	Solid	No	80	Taguchi et al. (13)
Male	17 years	Right	Circle or ellipse	Yes	Solid	Yes		Apoznanski et al. (14)
Male	3 years	Right	Circle or ellipse	Yes	Solid	Yes	180	Houben et al. (15)
Female	2 years	Left	Circle or ellipse	No	Solid	Yes	125	Houben et al. (16)
Male	2 years	Right	Circle or ellipse	Yes	Solid	No	100	Rojas et al. (4)
Female	4 years	Left	Circle or ellipse	No	Cystic solid	No	52	Case 1
Male	3 years	Left	Circle or ellipse	Yes	Solid	Yes	64	Case 2

EWT is often associated with the urinary system and spinal deformities. For instance, retroperitoneal EWT frequently coexists with horseshoe kidneys. The coexistence of EWT and horseshoe kidneys was first reported by Moyson in 1961, and several related cases have been reported since then. According to literature statistics, approximately 7.6% of EWT cases are accompanied by horseshoe kidneys (16). Furthermore, EWT in the lumbosacral spinal canal is often accompanied by spinal malformations and diastematomyelia. The mechanism of EWT in the spinal canal may be due to the differentiation of mesenchymal cells in the spine into nephrogenic components, which then undergo malignant transformation to produce EWT (17). Mirkin et al. (18) reported a case of a 2-year-old female patient with EWT. During surgery, a round cystic mass was found in the spinal canal, and the arch plates of T12-S2 near the mass were fused. The distance between the bilateral pedicles of L2 was significantly widened, and a bone spur originated to the left of the posterior midline of the fused L1/L2 arch plate, extending to the front of the mass. The mass was pathologically confirmed as EWT. Myelography revealed that the bone spur compressed the spinal cord, resulting in diastematomyelia from the upper edge of the T12 vertebral body to the L3–4 spinal cord. Similarly, Sharma et al. (19) reported a case of an 18-month-old female patient with EWT. The mass was located intradurally at the L2–L5 level, and a bone spur originated from the anterior edge of the first lumbar vertebral body, resulting in diastematomyelia. Retroperitoneal EWT can be associated with spinal deformities, but the specific mechanism is not clear. It is speculated that the residual retroperitoneal nephrogenic components undergo malignant transformation and convert to EWT and also stimulate the proliferation of adjacent vertebral bodies, resulting in spinal deformities. In another study, McCauley et al. (20) reported a case of a 4-year-old female patient with EWT. During surgery, a mass was found in the pelvic abdomen, and a bone spur originated from the anterior edge of the first sacrum and extended to the posterior edge of the mass. Similarly, in our hospital, we observed two patients with EWT, both of whom had a bone spur originating from the edge of the lumbar vertebral body and extending to the back of the tumors. In case 1, the position of the kidney on the ipsilateral side of the mass was lower, and its upper pole was at about the level of the L2 lumbar vertebrae. Furthermore, a left accessory renal artery entered the left renal artery from the beginning of the left iliac artery.

## Conclusion

In summary, due to the limited understanding of EWT, radiologists may not be able to provide an accurate diagnosis before the operation. Therefore, surgical exploration and pathological analysis are necessary for a definitive diagnosis. Based on the cases reported and previous literature, it can be observed that retroperitoneal EWT in children typically appears as a solid or cystic-solid mass on CT images. These masses are often large in size, have a clear boundary, and may exhibit calcification or a fat shadow within the mass. Moreover, the presence of a horseshoe kidney or spinal deformity can also aid in the diagnosis of EWT.

## Data Availability

The original contributions presented in the study are included in the article, further inquiries can be directed to the corresponding author.
